# Transcriptomic Profiling of Carboplatin- and Paclitaxel-Resistant Lung Adenocarcinoma Cells Reveals *CSF3* as a Potential Biomarker for the Carboplatin Plus Paclitaxel Doublet Regimens

**DOI:** 10.3390/cimb46120834

**Published:** 2024-12-11

**Authors:** Pritsana Raungrut, Suchanan Tanyapattrapong, Thipphanet Masjon, Saowanee Maungchanburi, Paramee Thongsuksai

**Affiliations:** 1 Division of Biomedical Sciences and Biomedical Engineering, Faculty of Medicine, Prince of Songkhla University, Hat Yai 90110, Songkhla, Thailand; bsuchanan.t@gmail.com (S.T.); thippanet9213@gmail.com (T.M.); msaowane@medicine.psu.ac.th (S.M.); 2 Department of Pathology, Faculty of Medicine, Prince of Songkhla University, Hat Yai 90110, Songkhla, Thailand; tparamee@gmail.com

**Keywords:** non-small cell lung cancer, carboplatin, paclitaxel, chemoresistance, transcriptomic profiling, biomarkers

## Abstract

This study aimed to generate Car- and Pac-resistant cell lines from the human lung adenocarcinoma H1792 cell line, designated as H1792/Car and H1792/Pac, and perform transcriptome sequencing to identify potential targets. Common differentially expressed genes (Co-DEGs) in both resistant cell lines were identified, followed by hub gene identification. Online validation was conducted through GEPIA and Kaplan–Meier Plotter platforms, with experimental validation performed using real-time quantitative PCR (RT-qPCR). After six months, the H1792/Car and H1792/Pac cell lines exhibited a 10.7-fold and 5.6-fold increase in resistance to Car and Pac, respectively. Flow cytometry analysis demonstrated that both resistant cell lines were resistant to cell cycle arrest and apoptosis induced by Car or Pac. Transcriptomic sequencing identified 123 Co-DEGs, including 72 upregulated and 51 downregulated genes, consistently expressed in both H1792/Car and H1792/Pac cell lines. Among these, 13 hub genes were identified, with colony-stimulating factor 3 (*CSF3*) uniquely associated with post-progression survival (PPS) in adenocarcinoma patients undergoing chemotherapy. Notably, *CSF3* expression was significantly elevated in both H1792/Car and H1792/Pac compared to parental cells. These findings underscore the value of drug-resistant models in uncovering critical biomarkers. *CSF3* emerges as a promising guiding marker or potential molecular target for optimizing Car- and Pac-based doublet regimens.

## 1. Introduction

Non-small cell lung cancer (NSCLC) accounts for approximately 85% of all lung cancer cases [1], with adenocarcinoma being the most common histological subtype, representing 40% of NSCLC cases [2]. More than 50% of patients are diagnosed with metastatic disease, which has a dismal 5-year survival rate of only 5% [3]. While novel targeted therapies and immunotherapies have the potential to improve overall survival in advanced NSCLC, these treatments are only applicable to a minority of patients. Consequently, the majority rely on chemotherapy as their primary treatment option. Chemotherapy can be administered either as a single agent (singlet) or as a combination of two agents (doublet). Meta-analyses have demonstrated that doublet chemotherapy is more effective than singlet chemotherapy in advanced NSCLC [4]. Several decades ago, third-generation platinum-based chemotherapy regimens, which combine platinum drugs such as cisplatin (Cis) or carboplatin (Car) with third-generation agents like gemcitabine (Gem) or paclitaxel (Pac), became the standard first-line treatment for advanced NSCLC patients without oncogenic driver mutations [5]. However, due to the significant toxicity associated with Cis, Car is often used as a substitute to mitigate these adverse effects [6]. A commonly used platinum-based regimen, Car plus Pac, achieves an initial response rate of approximately 40%, with a median survival time of 14.1 months. Unfortunately, the remaining patients often exhibit resistance to treatment due to the development of chemoresistance [7,8]. Chemoresistance involves multiple signaling pathways, including DNA repair mechanisms, pro-survival signaling, and drug efflux systems [9]. The underlying mechanisms may differ depending on whether a single drug or a combination of drugs is used. Despite extensive research to identify biomarkers that can guide treatment selection, the results remain inconsistent and inconclusive [10]. This highlights the need for models that can identify novel potential genes associated with chemotherapy response, particularly in the context of platinum-based regimens that mirror actual clinical treatments.

In this study, we aimed to establish two chemoresistant cell lines derived from the human H1792 adenocarcinoma cell line: carboplatin-resistant (H1792/Car) and paclitaxel-resistant (H1792/Pac) cells. We evaluated the cross-resistance of these cell lines to other chemotherapy drugs, including Cis, Car, Gem, and Pac, as well as to doublet regimens such as Car plus Pac, Car plus Gem, and Cis plus Gem. Transcriptome sequencing was used to identify differentially expressed genes (DEGs) between the resistant cell lines (H1792/Car and H1792/Pac) and their parental H1792 cells. We further explored and validated common differentially expressed genes (Co-DEGs) associated with resistance to both drugs. We believe that the chemoresistant H1792 cell lines developed in this study provide a valuable platform for preclinical models aimed at identifying and validating novel biomarkers. These biomarkers could help inform the selection of effective treatment regimens for advanced NSCLC patients, particularly those undergoing platinum-based chemotherapy.

## 2. Materials and Methods

### 2.1. Cell Line and Drugs

The parental H1792 cell line, derived from human lung adenocarcinoma, was purchased from the American Type Culture Collection (ATCC; Manassas, VA, USA). The cells were cultured in complete RPMI-1640 medium (Sigma-Aldrich, St. Louis, MO, USA) supplemented with 10% fetal bovine serum (FBS; Gibco, Thermo Fisher Scientific, Waltham, MA, USA) and 1% penicillin-streptomycin (5000 U/mL; Gibco). Cultures were maintained at 37 °C in a humidified incubator with 5% CO_2_. When cell confluence reached 80–90%, the cells were trypsinized using 0.25% trypsin-EDTA (Gibco), counted using a hemocytometer with 0.4% trypan blue staining (Gibco), and observed under a microscope. Pac, Car, Cis, and Gem were purchased from MedChemExpress (Monmouth Junction, NJ, USA), dissolved in distilled water, and stored at −20 °C according to the manufacturer’s instructions. Before use, aliquots were diluted to the required concentrations.

### 2.2. Generation of H1792/Car and H1792/Pac Cell Lines

**Figure 1:**
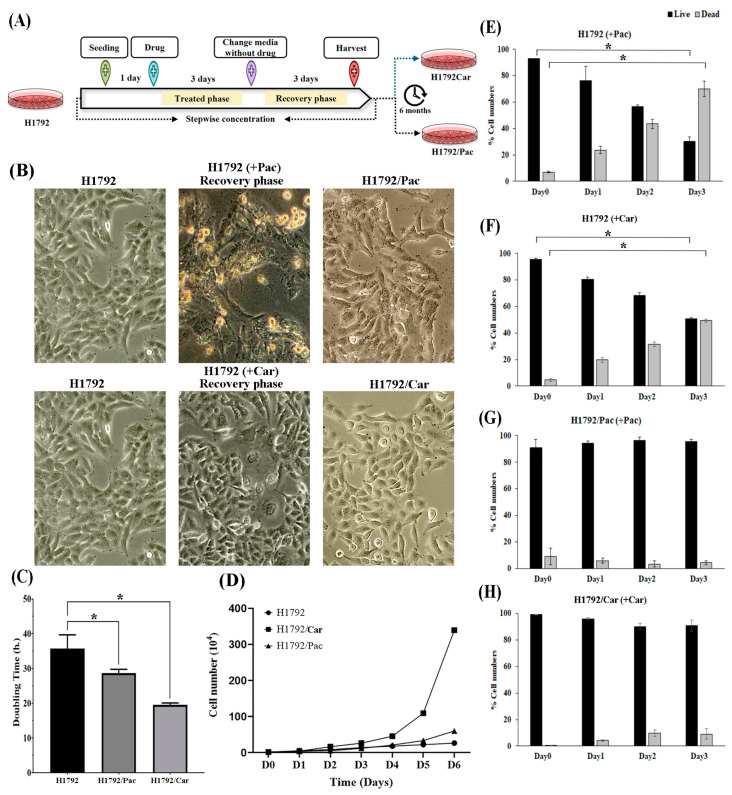
Resistance properties of drug-resistant cell lines. (**A**) Schematic representation of the experimental workflow used to generate H1792/Car and H1792/Pac cell lines using a stepwise method. (**B**) Morphological characteristics of parental H1792 cells, H1792 cells in the recovery phase post-treatment with Pac or Car at concentrations of 10 nM and 5 µM, respectively, and drug-resistant cells following recovery were observed under a microscope at 20× magnification. (**C**) Population doubling time of parental and drug-resistant cells. * Significant differences were determined using an independent *t*-test (*p* < 0.05). (**D**) Growth curves of parental and drug-resistant cells. (**E**) Live and dead cell counts following treatment of H1792 cells with 50 nM of Paclitaxel. (**F**) Live and dead cell counts following treatment of H1792 cells with 35 µM of Carboplatin. (**G**) Live and dead cell counts following treatment of H1792/Pac cells with 50 nM of Paclitaxel. (**H**) Live and dead cell counts following treatment of H1792/Car cells with 35 µM of Carboplatin. * Significant differences were determined using an ANOVA test (*p* < 0.05).

Drug-resistant cell lines were developed using a stepwise method, as described in a previous study [11]. Briefly, the IC_50_ concentrations (the concentration inhibiting 50% of cell growth) of Pac and Car for parental H1792 cells were used as the starting doses: 5 µM for Car and 10 nM for Pac. A total of 5 × 10^5^ cells were seeded into T25 flasks (Corning Inc., Corning, NY, USA) and incubated for 24 h. A complete medium containing either Pac or Car was added, and cells were incubated for 3 days (treatment phase). Following this, the drug-containing medium was replaced with a drug-free medium, and cells were cultured for an additional 3 days or until they reached a confluence of 70–80% (recovery phase). This cycle was repeated three times to confirm resistance. Subsequently, the drug concentration was gradually increased over a period of 6 months (Figure 1A). The resistance phenotype was validated by constructing dose–response curves for the resistant cells (H1792/Car and H1792/Pac) compared to parental H1792 cells. IC_50_ values were calculated, and the fold change (FC) in resistance was determined as the ratio of the IC_50_ of resistant cells to that of parental cells. Resistant cells were either used immediately in experiments or stored in liquid nitrogen until needed.

### 2.3. Analysis of Morphological Changes

Morphological changes in chemoresistant cells compared to parental cells were observed using an inverted microscope (Olympus IX71, Tokyo, Japan). Images of chemoresistant cells were captured and analyzed to identify any differences in cellular morphology.

### 2.4. 3-(4,5-Dimethylthiazol-2-yl)-2,5-diphenyltetrazolium Bromide (MTT) Assay

Cell viability following drug treatment was assessed using the MTT assay, which measures mitochondrial activity. Both single-agent treatments (Pac, Car, Cis, and Gem) and combination treatments (Car plus Pac, Car plus Gem, and Cis plus Gem) were evaluated for their effects on H1792, H1792/Pac, and H1792/Car cells. Cells were seeded at a density of 1 × 10^4^ cells per well in 96-well plates (Corning) and incubated for 24 h in a humidified atmosphere. For single-agent treatments, cells were exposed to Pac at concentrations of 0–1000 nM and to Car, Cis, and Gem at concentrations of 0–1000 µM. For combination treatments, the same concentration ranges were used for Car, Cis, and Gem, while Pac concentrations ranged from 0 to 1000 nM. After 3 days of drug exposure, MTT solution (final concentration: 0.5 µg/mL; Gibco) was added to each well and incubated for 2 h at 37 °C in a 5% CO_2_ incubator. Dimethyl sulfoxide (DMSO; Gibco) was then added, and the plates were incubated at room temperature for 30 min. The resulting colored solution was quantified by measuring absorbance at 570 nm (reference: 650 nm) using a microplate reader (Molecular Devices, San Jose, CA, USA). Each experiment was performed in duplicate and repeated at least twice. The IC_50_ values were calculated using an online IC_50_ calculator (https://www.aatbio.com/tools/ic50-calculator, accessed on 1 June 2023), and the resistance fold change was determined.

### 2.5. Growth Curve and Population Doubling Time

Parental H1792, H1792/Pac, and H1792/Car cells were trypsinized and seeded at a density of 2 × 10^4^ cells/well in duplicate in 12-well plates (Corning). Cells were collected and counted daily for six consecutive days using trypan blue staining. Growth curves were plotted, and the population doubling time was calculated using the following formula:

$$Doubling\;time=\frac{Duration\cdot ln(2)}{ln\left(\frac{Final\;concentration}{Initial\;concentration}\right)}$$

### 2.6. Trypan Blue Assay

Cells were seeded at a density of 2 × 10^4^ cells/well in triplicate in 6-well plates. After 24 h of incubation, parental H1792 cells were treated with 50 nM of Pac or 35 μM of Car. Similarly, H1792/Pac cells were treated with 50 nM of Pac, and H1792/Car cells were treated with 35 μM of Car. Following drug treatment, cells were trypsinized and resuspended in a complete medium for three consecutive days. Cell suspensions were mixed with 0.4% trypan blue solution at a 1:1 ratio. Dead cells were stained blue, while live cells remained unstained. The numbers of live and dead cells were counted under an inverted microscope. The proportion of cells was calculated using the formula: Cell number (%) = (Number of dead or live cells/Total number of cells) × 100.

### 2.7. Determination of Apoptosis by Flow Cytometry

Cell death mechanisms, including apoptosis and necrosis, were analyzed using flow cytometry. Parental and resistant cells (5 × 10^5^ cells) were seeded in T25 flasks and incubated for 24 h. Car was added at concentrations of 25 μM and 35 μM to H1792 and H1792/Car cells, respectively, while Pac was added at concentrations of 20 nM and 50 nM to H1792 and H1792/Pac cells, respectively. After 3 days of incubation at 37 °C in 5% CO_2_, cells were trypsinized, and 1 × 10^6^ cells were pelleted by centrifugation at 700× *g* for 5 min. The cells were washed with 400 µL of PBS and resuspended in 65 µL of 1× binding buffer. Annexin V-FITC (4 µL; BD Biosciences, San Jose, CA, USA) and propidium iodide (PI, 4 µL; BD Biosciences) were added, and the cells were incubated at room temperature for 30 min in the dark. Samples were analyzed using the Amnis^®^ ImageStream^®^X Mk II (Luminex Corporation, Austin, TX, USA). Data from Annexin V/PI staining were processed with IDEAS software version 6.5.0 (Luminex Corporation) to calculate the percentage of live and dead cells.

### 2.8. Determination of Cell Cycle by Flow Cytometry

The DNA content of cells was assessed using flow cytometry. Parental H1792 and H1792/Car cells were treated with Car at 25 μM and 35 μM, respectively, while H1792 and H1792/Pac cells were treated with Pac at 20 nM and 50 nM, respectively. After 3 days of incubation at 37 °C in 5% CO_2_, the media was removed, and the cells were incubated with new media containing 1% FBS for an additional 24 h to induce starvation. Cells were trypsinized and centrifuged at 700× *g* for 5 min. A total of 1 × 10^6^ cells were fixed in 1 mL of ice-cold 70% ethanol (JT Baker, Phillipsburg, NJ, USA) and stored overnight at −20 °C. The fixed cells were centrifuged, washed with 400 µL of PBS, and resuspended in 65 µL of 1× binding buffer. Subsequently, 100 µL of the cell suspension (2 × 10^5^ cells) was transferred to a new tube and mixed with 4 µL of PI (BD Biosciences) and 1 µL of RNase A (10 mg/mL; Vivantis, Selangor, Malaysia). After incubation at room temperature for 30 min in the dark, cell cycle distribution was analyzed using the Amnis^®^ ImageStream^®^X Mk II with IDEAS software (Luminex Corporation). The percentage of cells in each phase of the cell cycle was determined.

### 2.9. Transcriptome Sequencing

Total RNA was extracted using RNeasy Plus Mini Kits (Qiagen, Redwood City, CA, USA). RNA concentration and purity were measured using a NanoDrop^®^ ND-1000 UV–Vis spectrophotometer (Thermo Scientific), and RNA quality was assessed with a 2100 Bioanalyzer (Agilent Technologies, Rockville, MD, USA). Transcriptome sequencing was performed using the TruSeq mRNA Library Preparation Kit (Illumina Inc., San Diego, CA, USA) and a NovaSeq 6000 instrument (Illumina Inc.), following the manufacturer’s instructions.

Gene expression levels for each sample were estimated using the RSEM package. FastQC (https://www.bioinformatics.babraham.ac.uk/projects/fastqc/, all accessed on 25 July 2023) was used to generate quality-control reports. Low-quality reads and contamination from adaptors or primers were removed using the Trimmomatic program version 0.32 (http://www.usadellab.org/cms/?page=trimmomatic). Cleaned reads were aligned to the reference human genome using HTSeq program version 1.99.2 (https://htseq.readthedocs.io/en/master/). Gene expression levels were quantified in fragments per kilobase of transcript per million mapped reads (FPKM) using Cufflinks software version 2.2 (http://cole-trapnell-lab.github.io/cufflinks/manual/). Differentially expressed genes (DEGs) between parental H1792 cells and H1792/Car or H1792/Pac cells were identified using the DESeq2 R package in R (version 3.1.0). *p*-values were adjusted for multiple testing using the false discovery rate (FDR) method. Genes with an adjusted *p*-value < 0.05 and a Log_2_ FC ≥ 2 were considered significant.

### 2.10. Bioinformatics Analysis and Survival Analysis

Gene classifications were determined using ShinyGO 0.80 (http://bioinformatics.sdstate.edu/go77/). Gene Ontology (GO) and Kyoto Encyclopedia of Genes and Genomes (KEGG) pathway analyses of the DEGs were conducted using the Database for Annotation, Visualization, and Integrated Discovery (DAVID) (https://david.ncifcrf.gov/tools.jsp). Data visualization, including volcano plots, cluster heatmaps, and multi-group bubble plots, was performed using the Science and Research online plot tool (SR plot) (https://www.bioinformatics.com.cn/en). Venn diagrams illustrating Co-DEGs between H1792 vs. H1792/Car and H1792 vs. H1792/Pac cells were created using the Venny tool (https://bioinfogp.cnb.csic.es/tools/venny/).

Protein–protein interaction (PPI) network analysis was conducted using the STRING database, and the top 13 hub genes among the Co-DEGs were identified using Cytoscape version 3.10.2 with the CytoHubba plugin. Gene Expression Profiling Interactive Analysis version 2 (GEPIA2) (http://gepia2.cancer-pku.cn/#index) was utilized to evaluate gene expression levels. Kaplan–Meier Plotter (https://kmplot.com/analysis/) was employed to analyze post-progression survival (PPS), defined as the time from progression to death in adenocarcinoma patients treated with chemotherapy. The best cutoff was auto-selected, and HRs were calculated using the Cox proportional hazards (Cox PH) model.

### 2.11. Real-Time Quantitative Reverse Transcription PCR (RT-qPCR)

Total RNA was extracted from parental and resistant cells (1 × 10^6^ cells) using RNeasy Plus Mini Kits (Qiagen), following the manufacturer’s protocol. RNA quality and quantity were verified by agarose gel electrophoresis and a NanoDrop^®^ ND-1000 UV–Vis spectrophotometer (Thermo Scientific). Complementary DNA (cDNA) was synthesized from 1 µg of total RNA using a cDNA synthesis kit (Bio-Rad Laboratories, Hercules, CA, USA) on a Thermal Cycler (Bio-Rad Laboratories).

Target gene expression levels (*CSF3* and *IL1A*) were quantified using SYBR Green-based RT-qPCR. Specific primers were designed with Primer3Plus (https://www.bioinformatics.nl/cgi-bin/primer3plus/primer3plus.cgi) and used in triplicate reactions. The primer sequences were as follows: *IL1A*: forward 5′-TGTGACTGCCCAAGATGAAG-3′; reverse 5′-CGTGAGTTTCCCAGAAGAAGAG-3′; *CSF3*: forward 5′-CAACTCCATAGCGGCCTTT-3′; reverse 5′-CAGATGGTGGTGGCAAAGT-3′; *GAPDH* (endogenous control): forward 5′-CCACAGTCCATGCCATCAC-3′; reverse 5′-TCCACCACCCTGTTGCTGTA-3′.

Reactions were performed on a Thermal Cycler with the following conditions: initial denaturation at 95 °C for 2 min, followed by 39 cycles of 95 °C for 15 s and 64 °C for 30 s. Gene expression was calculated using the comparative cycle threshold (2^−ΔΔCt^) method, with *GAPDH* as the normalization control. Differences in expression between resistant and parental cells were analyzed using an unpaired *t*-test in Excel, with *p* < 0.05 considered statistically significant.

### 2.12. Statistical Analysis

Statistical analyses were conducted using GraphPad Prism 5.0 software (GraphPad Software, Inc., La Jolla, CA, USA). Experiments were performed in duplicate, and data were expressed as mean ± standard deviation (SD). A one-way ANOVA was used to analyze differences among multiple groups, while an independent *t*-test was employed for comparisons between two groups. Statistical significance was set at *p* < 0.05.

## 3. Results

### 3.1. Characterization of Drug-Resistant Cells

After a 6-month induction period, the resistant cell lines H1792/Car and H1792/Pac were successfully developed. Parental H1792 cells exhibited a typical polygonal morphology characteristic of epithelial cells. Following 3 days of treatment with Car or Pac, approximately 60–70% of the H1792 cells were eliminated. Significant morphological changes were observed during the recovery phase. After Pac treatment, H1792 cells displayed cytoplasmic shrinkage. In contrast, Car treatment resulted in an enlarged cell morphology, with a nucleus-to-cytoplasmic ratio of approximately 1:2, and the gradual emergence of mixed populations consisting of large and normal epithelial cells after 3–4 rounds of treatment. Both H1792/Car and H1792/Pac cells reverted to an epithelial-like morphology, confirming the successful establishment of drug-resistant cell lines (Figure 1B).

The growth rates of the resistant cells were reduced compared to the parental H1792 cells (Figure 1D). The doubling times of H1792/Car (19.50 h) and H1792/Pac (28.60 h) were significantly shorter than that of the parental H1792 cells (35.80 h), with a *p*-value < 0.001 (Figure 1C). After Pac (Figure 1E) and Car (Figure 1F) treatment, parental H1792 cells showed a time-dependent reduction in live cells and an increase in dead cells. In contrast, H1792/Pac (Figure 1G) and H1792/Car (Figure 1H) cells exhibited no significant changes in the proportions of live and dead cells following Car and Pac treatment, respectively.

### 3.2. Effect of Car and Pac on Cell Cycle and Apoptosis in Drug-Resistant Cells

**Figure 2:**
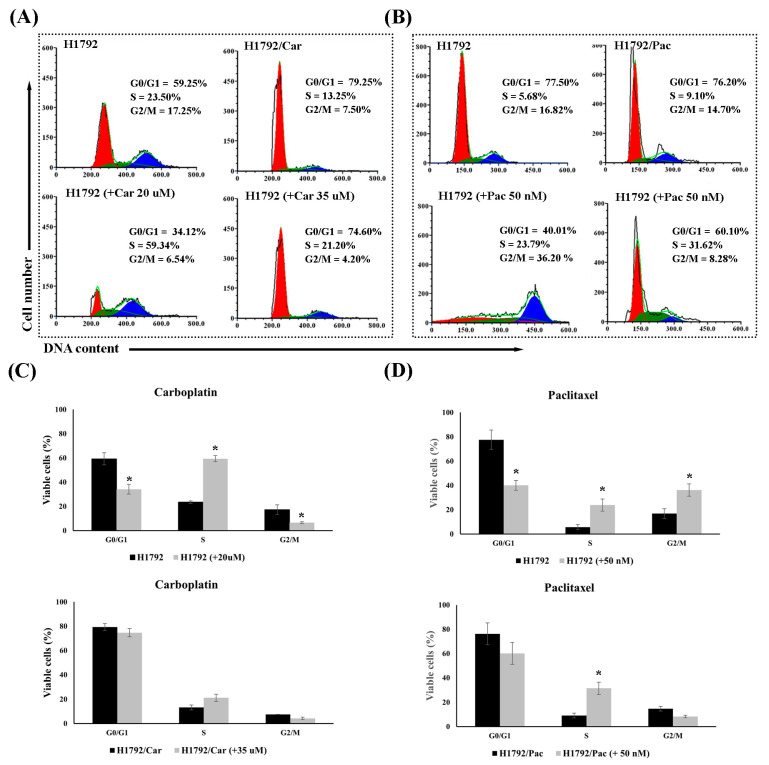
Cell cycle analysis by flow cytometry. (**A**) Cell cycle distribution in untreated H1792 and H1792/Car cells, and in H1792 and H1792/Car cells treated with Carboplatin at 20 µM and 35 µM, respectively. (**B**) Cell cycle distribution in untreated H1792 and H1792/Pac cells and in H1792 and H1792/Pac cells treated with Paclitaxel at 50 nM. (**C**) Representative flow cytometry histograms of cell cycle phases for H1792/Car cells under the indicated conditions. (**D**) Representative flow cytometry histograms of cell cycle phases for H1792/Pac cells under the indicated conditions. * Significant differences were determined using an independent *t*-test (*p* < 0.05).

The resistance properties of H1792/Car and H1792/Pac cells were further validated by analyzing cell cycle distribution and apoptosis using flow cytometry. In H1792 cells treated with 20 μM of Car, there was a significant increase in the S phase population (59.34% vs. 23.50%, *p* < 0.001) and a decrease in the G2/M phase population (6.54% vs. 17.25%, *p* = 0.032) compared to untreated cells. However, no significant changes in the cell cycle phases were observed in H1792/Car cells treated with 35 μM of Car compared to untreated H1792/Car cells (Figure 2A). Similarly, Pac treatment at 50 nM caused a significant increase in the G2/M phase (36.20% vs. 16.82%, *p* = 0.048) and S phase (23.79% vs. 5.68%, *p* < 0.001) in parental H1792 cells compared to untreated controls. In contrast, H1792/Pac cells treated with 50 nM of Pac showed only S phase arrest (31.62% vs. 9.10%, *p* = 0.023) compared to untreated H1792/Pac cells (Figure 2B).

**Figure 3:**
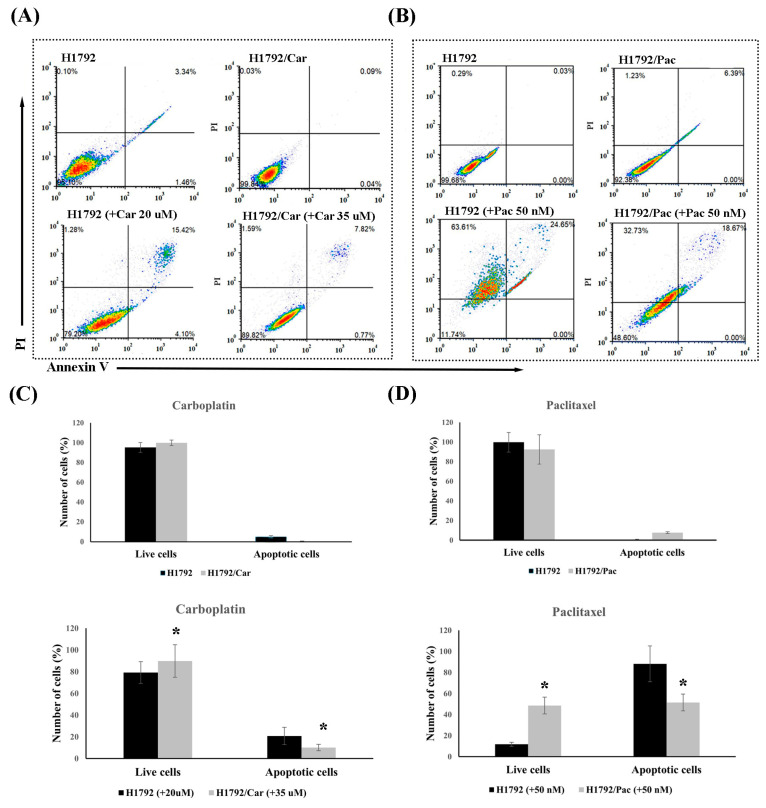
Cell apoptosis analysis by flow cytometry. (**A**) Apoptosis analysis of untreated H1792 and H1792/Car cells, and of H1792 and H1792/Car cells treated with Carboplatin at 20 µM and 35 µM, respectively. (**B**) Apoptosis analysis of untreated H1792 and H1792/Pac cells and of H1792 and H1792/Pac cells treated with Paclitaxel at 50 nM. (**C**) Representative flow cytometry plots of apoptotic cell populations in H1792/Car cells under the indicated conditions. (**D**) Representative flow cytometry plots of apoptotic cell populations in H1792/Pac cells under the indicated conditions. * Significant differences were determined using an independent *t*-test (*p* < 0.05).

Apoptosis was assessed using the Annexin-V-FITC/PI double staining assay. H1792/Car cells treated with 35 μM of Car demonstrated a higher percentage of live cells (89.82% vs. 79.20%, *p* = 0.042) and a lower percentage of apoptotic cells (10.18% vs. 20.80%, *p* = 0.022) compared to parental H1792 cells treated with 20 μM of Car (Figure 3A,C). Similarly, after treatment with 50 nM of Pac, H1792/Pac cells showed a significantly higher percentage of live cells (48.60% vs. 2.00%, *p* < 0.001) and a significantly lower percentage of apoptotic cells (51.40% vs. 88.26%, *p* = 0.035) compared to parental H1792 cells (Figure 3B,D). These findings confirm that H1792/Car and H1792/Pac cells exhibit resistance to Car and Pac treatment, respectively.

### 3.3. Resistance to Singlet and Doublet Chemotherapy in Drug-Resistant Cells

**Figure 4:**
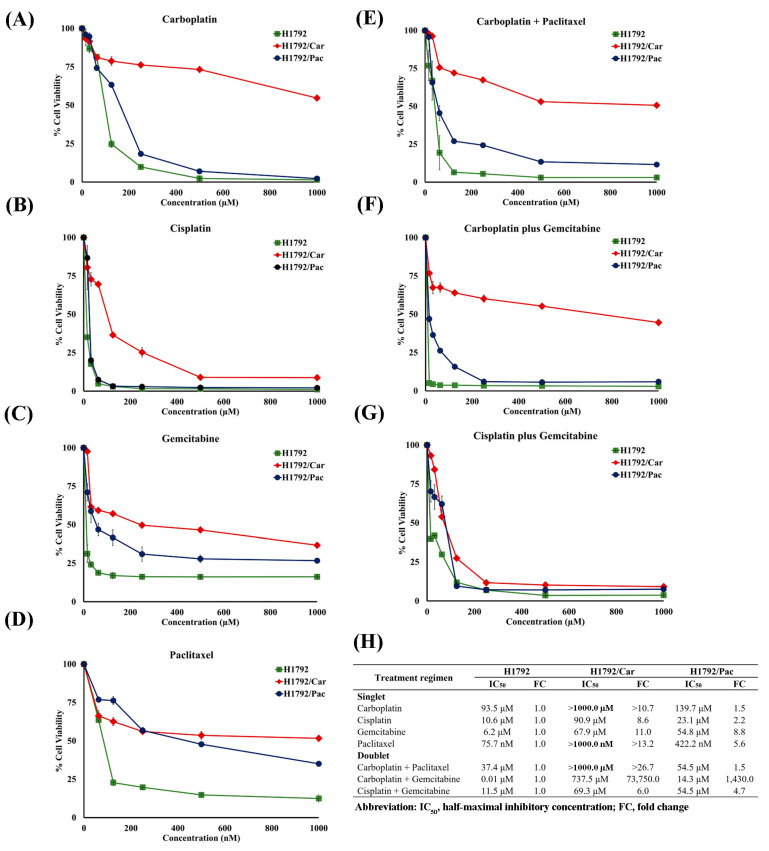
Cell viability analysis using MTT assay. (**A**–**D**) Cell viability of H1792, H1792/Car, and H1792/Pac cells following treatment with single agents: (**A**) Carboplatin, (**B**) Cisplatin, (**C**) Gemcitabine, and (**D**) Paclitaxel. (**E**–**G**) Cell viability following combination treatments: (**E**) Carboplatin plus Paclitaxel, (**F**) Carboplatin plus Gemcitabine, and (**G**) Cisplatin plus Gemcitabine. (**H**) Comparison of IC50 values for drug-resistant cells (H1792/Car and H1792/Pac) and parental H1792 cells after single-agent and combination chemotherapy.

In singlet chemotherapy, parental H1792 cells demonstrated sensitivity to Car, Cis, Gem, and Pac, with IC50 values of 93.5 µM, 10.6 µM, 6.2 µM, and 75.7 nM, respectively. In contrast, the H1792/Car and H1792/Pac cell lines, which were continuously exposed to Car and Pac, exhibited significant resistance. H1792/Car displayed a 10.7-fold increase in resistance to Car (Figure 4A), while H1792/Pac showed a 5.6-fold increase in resistance to Pac (Figure 4D). Additionally, H1792/Car exhibited cross-resistance to all tested chemotherapeutic agents, with resistance levels exceeding 8-fold for each drug (Figure 4B–D,H). H1792/Pac cells demonstrated cross-resistance to Cis and Gem, with FCs of 2.2 and 8.8, respectively (Figure 4A–C,H).

In doublet chemotherapy, the Car plus Gem regimen showed exceptionally high resistance, with H1792/Car and H1792/Pac cells exhibiting fold changes of 73,750 and 1430, respectively (Figure 4F,H). Resistance to the Cis plus Gem regimen was observed in both H1792/Car and H1792/Pac cells, with a 6-fold and 4.7-fold increase, respectively (Figure 4G,H). Resistance to the Car plus Pac regimen was notable only in H1792/Car cells, which displayed a fold change exceeding 26.7 (Figure 4E,H).

### 3.4. Identification of DEGs Between Parental and Resistant Cell Lines

**Figure 5:**
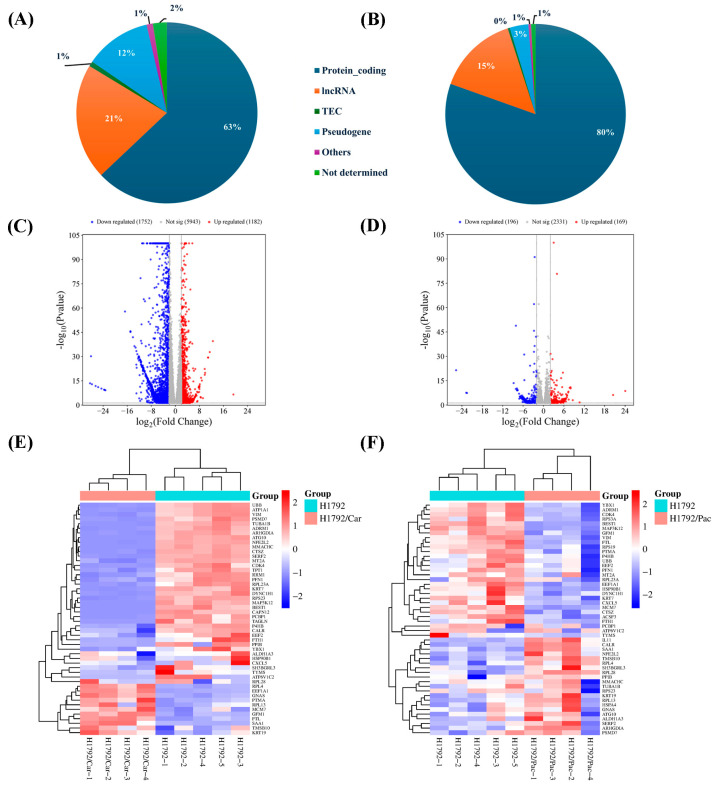
Identification of differentially expressed genes (DEGs) using transcriptomic sequencing. (**A**) Distribution of RNA types identified in H1792 and H1792/Car cells. (**B**) Distribution of RNA types identified in H1792 and H1792/Pac cells. (**C**,**D**) Volcano plots of DEGs for H1792/Car (**C**) and H1792/Pac (**D**) cells. (**E**,**F**) Clustered heat maps of DEGs for H1792/Car (**E**) and H1792/Pac (**F**) cells.

Transcriptomic analysis identified 14,105 genes with a *p*-value < 0.05 in the H1792 vs. H1792/Car comparison and 3351 genes in the H1792 vs. H1792/Pac comparison. Of these, protein-coding genes constituted 63% (8877 of 14,105 total RNA types) in the H1792 vs. H1792/Car comparison and 80% (2696 of 3351 total RNA types) in the H1792 vs. H1792/Pac comparison (Figure 5A,B). For downstream analyses, only protein-coding genes were selected.

Using the criteria of log2 FC ≥ 2 and adjusted *p*-value < 0.05, the analysis revealed 2934 DEGs in the H1792 vs. H1792/Car comparison, comprising 1182 upregulated and 1752 downregulated genes (Figure 5C). In the H1792 vs. H1792/Pac comparison, 365 DEGs were identified, with 169 upregulated and 196 downregulated genes (Figure 5D). Heatmaps displaying the top 50 DEGs for each comparison highlighted significant differences in gene expression profiles (Figure 5E,F). The most significant 50 DEGs (both upregulated and downregulated) for each comparison are detailed in Table 1.

### 3.5. Integrative Bioinformatics and Enrichment Analysis

**Figure 6:**
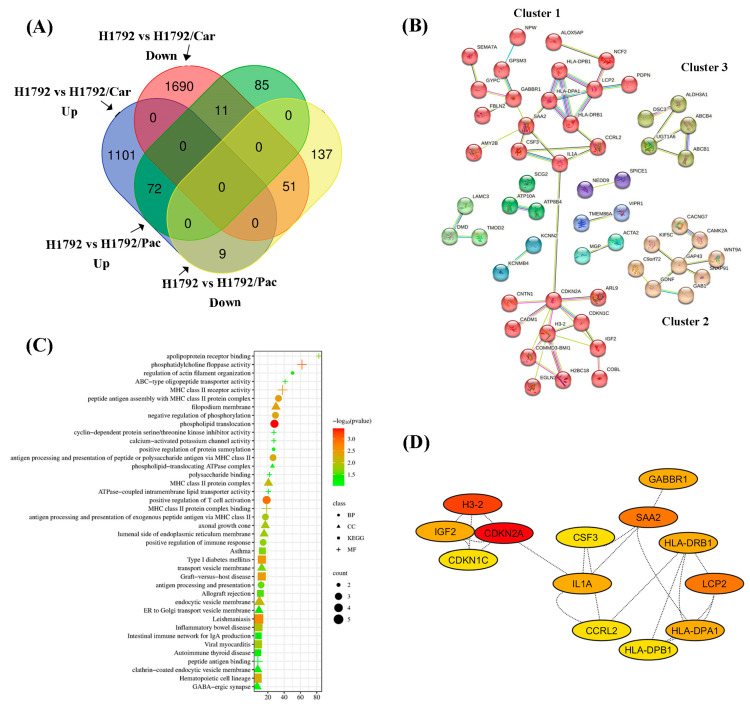
Identification of common DEGs (Co-DEGs) in drug-resistant cell lines. (**A**) Venn diagram illustrating 72 upregulated Co-DEGs and 51 downregulated Co-DEGs shared between H1792/Car and H1792/Pac datasets. (**B**) Cluster analysis of Co-DEGs using STRING. (**C**) Gene Ontology (GO) classification and KEGG pathway enrichment analysis of Co-DEGs. (**D**) Identification of hub genes based on degree ranking using the cytoHubba plug-in.

To identify Co-DEGs with similar directional patterns in both resistant cell lines, we integrated the upregulated and downregulated DEGs from each comparison using Venn diagram analysis. A total of 123 Co-DEGs were identified as consistent in both H1792/Car and H1792/Pac cells compared to the parental H1792 cells, including 72 upregulated and 51 downregulated genes (Figure 6A). The complete lists of consistent Co-DEGs are provided in Table 2 (upregulated) and Table 3 (downregulated). Notably, novel proteins were identified among these DEGs, including two upregulated genes and four downregulated genes. PPI network analysis of the Co-DEGs revealed 117 nodes and 60 edges. K-means clustering divided the network into three main clusters (Figure 6B). Cluster 1, containing 29 genes, was enriched for processes such as the positive regulation of response to stimuli, regulation of the immune system, and regulation of cell population proliferation. Cluster 2, comprising 9 genes, was associated with neuron differentiation, while Cluster 3 included 5 genes linked to phosphatidylethanolamine flippase activity.

GO enrichment analysis identified key biological processes, cellular components, and molecular functions. The most enriched biological processes were phospholipid translocation and T-cell activation. Cellular components were significantly enriched in the filopodium membrane and the MHC class II protein complex, while molecular functions highlighted MHC class II receptor activity (Figure 6C). KEGG pathway analysis revealed significant enrichment in pathways related to leishmaniasis and Type I diabetes mellitus (Figure 6C and Figure S1). Using Cytoscape 3.10.2 and its degree mode, 13 hub genes were identified within the PPI network (Figure 6D). Of these, 11 genes were upregulated (*LCP2*, *CSF3*, *IL1A*, *CCRL2*, *CDKN1C*, *SAA2*, *IGF2*, *HLA-DRB1*, *GABBR1*, *HLA-DPB1*, and *HLA-DPA1*), while 2 genes were downregulated (*H3-7* and *CDKN2A*) in drug-resistant cell lines compared to parental cells.

### 3.6. Hub Gene Screening and Survival Analysis

**Figure 7:**
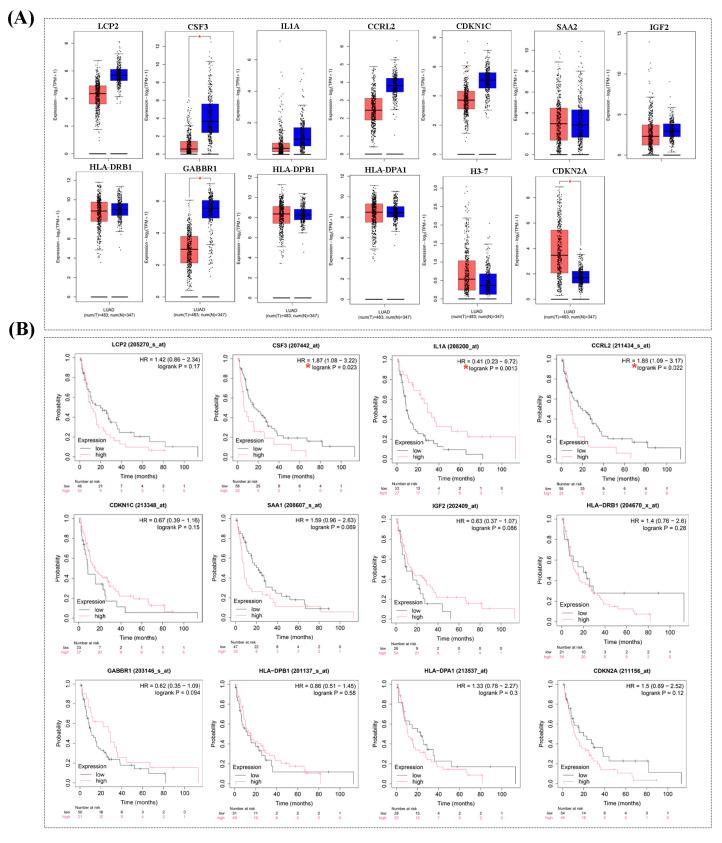
Gene expression and survival analysis. (**A**) Differential expression of 13 hub genes in lung adenocarcinoma compared to normal tissues, analyzed using the GEPIA2 database. (**B**) Post-progression survival (PPS) analysis of the 13 hub genes using the Kaplan–Meier Plotter. (*) indicates statistically significant differences compared to normal tissues (*p* < 0.05).

**Figure 8:**
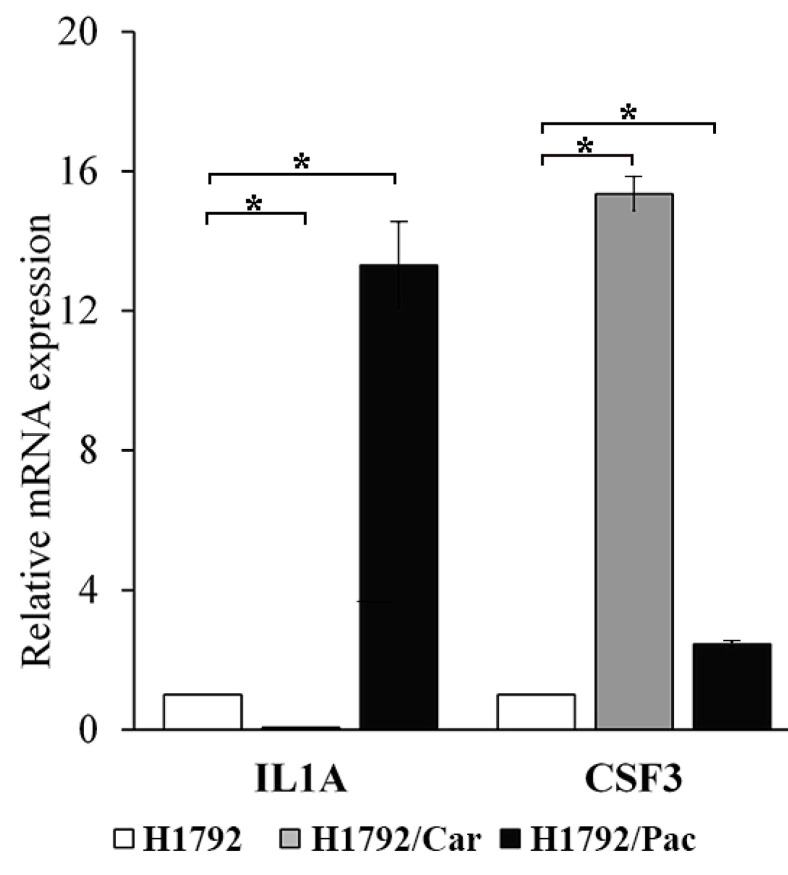
Validation of *IL1A* and *CSF3* gene expression via RT-qPCR. Gene expression levels of *IL1A* and *CSF3* in drug-resistant cell lines (H1792/Car and H1792/Pac) compared to parental H1792 cells. * Significant differences were determined using an independent *t*-test (*p* < 0.05).

The expression levels of the 13 hub genes were further analyzed using GEPIA2 in normal and lung tumor tissues. Among these, *CSF3* and *GABBR1* showed significantly reduced expression in lung adenocarcinoma tissues compared to normal tissues. In contrast, *CDKN2A* showed elevated expression in cancer tissues (Figure 7A). Prognostic analysis using the Kaplan–Meier Plotter revealed that higher expression of *CSF3* and *CCRL2*, as well as lower expression of *IL1A*, were significantly associated with prolonged PPS in adenocarcinoma patients undergoing chemotherapy (Figure 7B). To validate these findings, *CSF3* and *IL1A* were evaluated by RT-qPCR in the drug-resistant cell lines. The results showed a significant increase in *CSF3* expression in both H1792/Car and H1792/Pac cells. However, *IL1A* expression varied, with lower levels in H1792/Car cells and higher levels in H1792/Pac cells compared to the parental H1792 cells (Figure 8). Collectively, our findings suggest that increased *CSF3* expression may play a critical role in lung carcinogenesis and resistance to chemotherapy, particularly to Car and Pac.

## 4. Discussion

Drug response is a critical factor in the treatment of advanced NSCLC patients undergoing platinum-based chemotherapy due to the limited efficacy observed across various doublet regimens [7,12]. This variability is likely influenced by distinct mechanisms of drug response and resistance associated with specific chemotherapy agents and regimens. Developing drug-resistant cell lines that mimic clinical therapy conditions provides a foundation for identifying novel genes and pathways involved in chemotherapy response. Although the combination of Car and Pac is commonly used in advanced NSCLC, no drug-resistant cell lines have previously been generated in vitro for this doublet regimen.

In this study, we successfully established and characterized H1792/Car and H1792/Pac drug-resistant cell lines. These resistant cells were developed over six months through cyclic and incremental drug exposure. Morphological changes were observed during the induction process, and the growth rate and doubling time of the resistant cells were lower than those of the parental H1792 cells. Previous studies have shown that chemoresistant cells often exhibit an FC in resistance ranging from 5 to 10. For example, Hellweg et al. (2018) reported an 8-fold and 10-fold reduction in sensitivity to Car and Pac in endometrial cancer cells, respectively [13]. Similarly, Fu et al. (2018) found a 10.3-fold increase in Pac resistance in gastric carcinoma cells [14]. However, higher resistance levels have also been reported, such as a 100-fold reduction in sensitivity to Pac in ovarian cancer cells [15]. Our findings align with Hellweg et al. (2018) and Fu et al. (2018), showing that H1792/Car cells exhibited a 10.7-fold increase in resistance to Car, while H1792/Pac cells demonstrated a 5.6-fold increase in resistance to Pac. In clinical settings, dose escalation of chemotherapy is not routinely practiced due to potential adverse effects, making extreme levels of resistance unlikely in patients with advanced NSCLC. Thus, we consider the resistance levels observed in our drug-resistant cell lines to be representative and suitable for use as models in preclinical studies.

Recent strategies to enhance chemotherapy efficacy have focused on addressing cross-drug resistance. Efferth et al. (2008) revealed that tumors resistant to doxorubicin often exhibited resistance to other cytotoxic agents [16]. Similarly, Pac-resistant gastric carcinoma cells displayed cross-resistance to 5-fluorouracil and adriamycin [14], while taxol-resistant lung adenocarcinoma cells showed resistance to taxotere, colchicine, adriamycin, and bleomycin [17]. Cross-resistance to Pac has also been observed in Car-resistant ovarian cancer cells [18,19]. However, some studies reported no cross-resistance between Pac and Car in endometrial cancer cells [13]. Our results are consistent with most previous studies, demonstrating that H1792/Car and H1792/Pac cells exhibited cross-resistance to Cis, Gem, and Pac. Notably, we observed that resistance to a single drug also conferred resistance to combination treatments. For example, H1792/Car cells displayed a high degree of resistance to all doublet regimens, particularly the combination of Car plus Gem. This suggests that cross-resistance can occur in both singlet and doublet chemotherapy, likely due to mechanisms such as enhanced drug efflux, increased DNA repair capacity, and genetic alterations [8,16]. Interestingly, our findings suggest that the combination of Cis plus Gem may be more effective than other regimens, as it showed lower resistance levels compared to alternatives. However, to mitigate Cis-induced toxicity, the combination of Car plus Pac remains an essential treatment option for advanced NSCLC patients.

As a result, we further identified genes associated with resistance to the doublet regimen of Car and Pac. Through integrative analysis and bioinformatics tools, we determined that colony-stimulating factor 3 (*CSF3*) was upregulated in both drug-resistant cell lines. Additionally, elevated expression of *CSF3* correlated with the PPS of adenocarcinoma patients receiving chemotherapy. The gene expression level of *CSF3* was significantly higher in both H1792/Car and H1792/Pac cells compared to their parental counterparts. The *CSF3* gene encodes granulocyte colony-stimulating factor (G-CSF), a cytokine that regulates the proliferation, differentiation, and mobilization of neutrophils from the bone marrow [20]. G-CSF plays a pivotal role in the regulation of various immune functions [21] and is primarily expressed in myeloid cells [22]. In recent years, G-CSF has been widely recommended as an adjuvant treatment for severe cases of chemotherapy-induced neutropenia [23,24]. However, while G-CSF is therapeutically beneficial, it is also highly expressed in several tumors. Numerous studies have demonstrated that G-CSF can promote tumor metastasis, angiogenesis, and cancer proliferation by enhancing the immunosuppressive environment within the tumor microenvironment [25,26,27]. Increased G-CSF expression has been associated with poor overall survival in patients with triple-negative breast cancer [28], NSCLC [29], and cervical cancer [30].

Our findings are supported by previous studies, indicating that *CSF3* expression, and consequently the production of G-CSF, is associated not only with tumor progression but also with chemoresistance in NSCLC patients treated with the Car plus Pac doublet regimen. Consequently, these findings suggest that G-CSF may serve as a possible biomarker for cancer prognosis and could act as a predictive biomarker for treatment regimen selection in advanced NSCLC. Nevertheless, our current investigation is limited by the lack of tumor tissue samples from patients who received this specific doublet regimen. Furthermore, the functions of CSF3 regarding chemotherapeutic response remain unexplored. Consequently, additional functional studies in both parental H1792 and resistant cells, H1792/Car and H1792/Pac, as well as clinical studies in tissue samples, are necessary to validate the role of *CSF3* as a biomarker in this context.

## 5. Conclusions

We successfully established drug-resistant cell lines, H1792/Car and H1792/Pac, specific to the Car plus Pac regimen. This finding may validate earlier research in other cancers that employed the creation of drug-resistant cell lines as a model for identifying potential biomarkers for predicting response. Our findings indicate that resistance to a single drug also results in resistance to combination treatment. In addition, our transcriptomic sequencing, bioinformatics analysis, and technical validation of gene expression revealed a marked increase in *CSF3* expression in both cell lines. These findings suggest that *CSF3* may serve as a novel biomarker for resistance to the Car plus Pac regimen and a potential molecular target for addressing chemoresistance in advanced NSCLC patients.

## Figures and Tables

**Table 1:** Top 40 lists of DEGs between H1792 vs. H1792/Car and H1792 vs. H1792/Pac.

**H1792 vs. H1792/Car**					
**Upregulated DEGs**			**Downregulated DEGs**		
**Gene**	**log2FC**	***p*** **-adj**	**Gene**	**log2FC**	***p*** **-adj**
INO80B-WBP1	19.33	2.9 × 10^−7^	novel protein	−28.36	3.6 × 10^−14^
SOD2	12.49	3.0 × 10^−40^	novel protein	−28.06	6.6 × 10^−31^
novel transcript	11.46	1.7 × 10^−33^	novel protein	−27.73	1.3 × 10^−13^
WDR83OS	10.92	4.5 × 10^−30^	novel protein	−26.51	1.6 × 10^−12^
HLA-DRA	10.90	3.7 × 10^−30^	novel protein	−25.66	8.4 × 10^−12^
VIPR1	9.92	1.2 × 10^−20^	novel protein	−24.58	6.4 × 10^−11^
INHBA	9.80	1.9 × 10^−19^	PDCD6-AHRR	−23.70	3.2 × 10^−10^
ARPIN-AP3S2	9.79	4.7 × 10^−23^	novel protein	−23.52	4.4 × 10^−10^
novel protein	9.20	5.3 × 10^−5^	PCDHGA12	−23.25	7.1 × 10^−10^
novel protein	8.82	6.9 × 10^−16^	novel protein	−16.74	1.4 × 10^−58^
VARS2	8.56	1.4 × 10^−17^	novel transcript	−14.94	2.3 × 10^−46^
SPICE1	8.49	2.4 × 10^−5^	RPSAP58	−14.93	6.4 × 10^−46^
HLA-DRB1	8.41	1.2 × 10^−5^	SYNC	−14.90	3.3 × 10^−46^
ECE2	8.14	1.3 × 10^−13^	LHFPL5	−14.11	1.2 × 10^−42^
novel protein	8.05	4.6 × 10^−12^	novel protein	−13.60	4.0 × 10^−37^
FGF11	8.03	1.2 × 10^−10^	novel protein	−13.21	3.0 × 10^−36^
novel protein	8.01	1.1 × 10^−12^	LRTOMT	−12.97	1.0 × 10^−25^
C9orf72	7.95	8.2 × 10^−9^	PRRC2B	−12.95	4.2 × 10^−34^
novel protein	7.94	1.1 × 10^−11^	novel protein	−12.93	5.9 × 10^−8^
HOMEZ	7.92	2.8 × 10^−12^	TBCE	−12.86	8.8 × 10^−24^
**H1792 vs. H1792/Pac**					
**Upregulated DEGs**			**Downregulated DEGs**		
**Gene**	**log2FC**	***p*** **-adj**	**Gene**	**log2FC**	***p*** **-adj**
HNRNPUL2-BSCL2	24.06	2.18 × 10^−9^	novel protein	−25.72	3.62 × 10^−22^
INO80B-WBP1	20.47	6.62 × 10^−7^	PDCD6-AHRR	−22.73	2.76 × 10^−8^
novel protein	10.61	2.41 × 10^−2^	novel protein	−22.52	3.90 × 10^−8^
novel protein	8.61	1.02 × 10^−3^	LVRN	−8.79	3.09 × 10^−14^
SIRPB1	7.99	5.63 × 10^−11^	SMPDL3B	−8.14	1.17 × 10^−10^
INHBA	7.95	1.11 × 10^−11^	ALDH3A1	−8.13	1.41 × 10^−49^
VIPR1	7.70	2.45 × 10^−11^	LUM	−7.81	5.98 × 10^−11^
novel protein	7.41	4.19 × 10^−4^	HHIPL2	−7.81	9.06 × 10^−10^
LDB2	7.33	5.17 × 10^−9^	APOBEC3G	−7.67	8.91 × 10^−10^
PDPN	7.28	1.90 × 10^−8^	DCN	−7.59	6.12 × 10^−10^
PPP4R3C	6.97	7.14 × 10^−7^	GDNF	−7.19	1.05 × 10^−7^
TMEM265	6.88	2.35 × 10^−6^	KCNE3	−7.13	1.21 × 10^−4^
BTBD11	6.87	1.83 × 10^−5^	PYCARD	−6.97	5.48 × 10^−6^
NGF	6.63	1.87 × 10^−7^	MYB	−6.87	9.08 × 10^−6^
CSMD2	6.61	1.36 × 10^−4^	PCSK9	−6.83	5.09 × 10^−5^
SPICE1	6.60	6.16 × 10^−3^	MKX	−6.70	1.21 × 10^−5^
TIE1	6.46	4.09 × 10^−4^	VWCE	−6.60	1.84 × 10^−6^
LCP2	6.46	1.12 × 10^−4^	HTD2	−6.53	4.99 × 10^−5^
CNBD1	6.45	1.93 × 10^−4^	COL1A2	−6.38	4.09 × 10^−5^
MPZL2	6.35	1.01 × 10^−4^	ATP10A	−6.26	2.77 × 10^−4^

Abbreviations: FC, fold change; DEGs, differentially expressed genes; H1792/Car, Car-resistant H1792 cell line; H1792/Pac, Pac-resistant H1792 cell line.

**Table 2:** Lists of 72 Co-upregulated DEGs between H1792 vs. H1792/Car and H1792 vs. H1792/Pac.

Gene	H1792 vs. H1792/Car		H1792 vs. H1792/Pac		Gene	H1792 vs. H1792/Car		H1792 vs. H1792/Pac	
	log2FC	*p*-adj	log2FC	*p*-adj		log2FC	*p*-adj	log2FC	*p*-adj
ABCB4	4.01	1.88 × 10^−6^	3.29	9.34 × 10^−4^	MPZL2	5.24	3.53 × 10^−4^	6.35	1.01 × 10^−4^
CNTN1	5.08	4.11 × 10^−4^	4.69	6.69 × 10^−3^	TMEM86A	2.84	9.35 × 10^−3^	3.14	1.69 × 10^−2^
CLXN	5.02	8.55 × 10^−4^	5.80	7.09 × 10^−4^	BTBD11	4.34	3.75 × 10^−3^	6.87	1.83 × 10^−5^
RIPOR3	2.62	5.65 × 10^−8^	3.29	1.33 × 10^−10^	PTPRO	3.79	1.36 × 10^−5^	2.84	8.93 × 10^−3^
LCP2	4.15	7.16 × 10^−3^	6.46	1.12 × 10^−4^	PDPN	5.83	1.40 × 10^−6^	7.28	1.90 × 10^−8^
CDON	3.27	5.40 × 10^−4^	2.83	1.44 × 10^−2^	SPICE1	8.49	2.41 × 10^−5^	6.60	6.16 × 10^−3^
SNAP91	5.71	1.41 × 10^−4^	5.06	5.01 × 10^−3^	MYORG	2.73	1.60 × 10^−2^	2.80	4.77 × 10^−2^
CAMK2A	5.47	6.30 × 10^−4^	5.78	1.79 × 10^−3^	VSTM4	2.34	3.69 × 10^−2^	3.26	1.27 × 10^−2^
KCNN2	2.83	7.36 × 10^−3^	4.60	5.90 × 10^−5^	SERPINB7	4.33	2.22 × 10^−2^	6.18	4.02 × 10^−3^
ABCB1	2.21	4.53 × 10^−3^	3.68	1.08 × 10^−5^	IGF2	2.03	1.58 × 10^−5^	3.52	4.23 × 10^−13^
CYTH4	2.79	4.62 × 10^−2^	3.42	4.62 × 10^−2^	ZNF558	3.86	1.21 × 10^−14^	2.25	1.47 × 10^−4^
RASD2	3.58	1.95 × 10^−4^	2.67	2.71 × 10^−2^	KIF5C	4.45	3.63 × 10^−12^	3.37	3.48 × 10^−6^
TRIM9	4.28	2.93 × 10^−3^	5.99	1.52 × 10^−4^	LDB2	5.37	6.11 × 10^−6^	7.33	5.17 × 10^−9^
SIRPB1	4.51	1.67 × 10^−4^	7.99	5.63 × 10^−11^	SCG2	3.35	1.79 × 10^−6^	2.68	1.09 × 10^−3^
CACNG7	2.56	1.65 × 10^−16^	2.68	4.06 × 10^−16^	DMRTA1	6.11	3.12 × 10^−4^	5.54	6.21 × 10^−3^
FSD1L	3.71	4.60 × 10^−4^	2.76	4.14 × 10^−2^	ZNF443	4.65	1.40 × 10^−3^	4.83	4.73 × 10^−3^
CSF3	2.33	1.32 × 10^−4^	5.19	2.73 × 10^−17^	CADM1	3.31	1.07 × 10^−6^	2.53	1.59 × 10^−3^
GAB1	3.78	4.27 × 10^−5^	2.39	4.82 × 10^−2^	HLA-DRB1	8.41	1.16 × 10^−5^	5.69	1.62 × 10^−2^
VIPR1	9.92	1.23 × 10^−20^	7.70	2.45 × 10^−11^	ZNF836	2.43	1.23 × 10^−2^	2.96	9.55 × 10^−3^
IL1A	2.41	1.32 × 10^−12^	2.09	2.72 × 10^−8^	RFX8	3.93	2.86 × 10^−11^	3.08	3.75 × 10^−6^
RIMS3	5.14	1.56 × 10^−3^	5.63	2.83 × 10^−3^	ZNF33B	4.63	4.31 × 10^−4^	4.35	5.50 × 10^−3^
GDA	3.18	3.77 × 10^−4^	3.66	3.09 × 10^−4^	H2BC18	2.30	2.81 × 10^−2^	3.30	6.21 × 10^−3^
CCRL2	5.71	1.04 × 10^−4^	3.99	3.98 × 10^−2^	GABBR1	4.84	1.61 × 10^−17^	2.14	3.13 × 10^−3^
INHBA	9.80	1.90 × 10^−19^	7.95	1.11 × 10^−11^	VGLL3	5.41	5.37 × 10^−4^	5.45	2.97 × 10^−3^
PAIP2B	2.32	2.64 × 10^−3^	2.34	1.23 × 10^−2^	GPSM3	5.08	4.69 × 10^−5^	3.90	1.21 × 10^−2^
TMOD2	3.89	2.59 × 10^−7^	3.03	6.29 × 10^−4^	HLA-DPB1	4.19	3.47 × 10^−5^	5.56	3.30 × 10^−7^
EGLN3	3.05	1.99 × 10^−5^	2.56	2.47 × 10^−3^	PPP4R3C	4.52	6.72 × 10^−4^	6.97	7.14 × 10^−7^
CDKN1C	3.17	9.27 × 10^−3^	4.03	4.07 × 10^−3^	HLA-DPA1	4.18	1.26 × 10^−13^	3.33	1.47 × 10^−7^
GMFG	3.16	1.79 × 10^−3^	3.23	7.53 × 10^−3^	AMY2B	4.78	3.22 × 10^−3^	4.13	4.81 × 10^−2^
ALOX5AP	7.08	1.60 × 10^−6^	4.67	1.17 × 10^−2^	Novel protein	9.20	5.28 × 10^−5^	8.61	1.02 × 10^−3^
SAA2	3.57	1.57 × 10^−5^	3.90	2.25 × 10^−5^	Novel protein	5.13	6.73 × 10^−3^	7.41	4.19 × 10^−4^
SPX	4.72	2.94 × 10^−3^	4.67	1.58 × 10^−2^	COMMD3-BMI1	7.53	1.47 × 10^−8^	5.30	8.11 × 10^−4^
DSC3	3.45	6.28 × 10^−5^	4.91	9.74 × 10^−8^	INO80B-WBP1	19.33	2.93 × 10^−7^	20.47	6.62 × 10^−7^
KCNMB4	2.70	2.27 × 10^−4^	2.98	3.66 × 10^−4^	TMEM265	6.84	2.02 × 10^−7^	6.88	2.35 × 10^−6^
GYPC	4.94	2.47 × 10^−3^	6.18	7.96 × 10^−4^	PPFIA4	3.47	2.29 × 10^−4^	2.66	2.44 × 10^−2^
SEMA7A	2.05	1.85 × 10^−19^	2.23	7.67 × 10^−21^	C9orf72	7.95	8.21 × 10^−9^	5.65	4.96 × 10^−4^

Abbreviations: FC, fold change; DEGs, differentially expressed genes; H1792/Car, Car-resistant H1792 cell line; H1792/Pac, Pac-resistant H1792 cell line.

**Table 3:** Lists of 51 Co-downregulated DEGs between H1792 vs. H1792/Car and H1792 vs. H1792/Pac.

Gene	H1792 vs. H1792/Car		H1792 vs. H1792/Pac		Gene	H1792 vs. H1792/Car		H1792 vs. H1792/Pac	
	log2FC	*p*-adj	log2FC	*p*-adj		log2FC	*p*-adj	log2FC	*p*-adj
GAP43	−2.39	6.00 × 10^−3^	−4.68	2.53 × 10^−3^	HHIPL2	−3.32	3.75 × 10^−8^	−7.81	9.06 × 10^−10^
HSPB6	−4.32	3.25 × 10^−10^	−3.57	3.90 × 10^−6^	ARL9	−2.44	3.39 × 10^−7^	−2.74	1.05 × 10^−6^
SMPDL3B	−4.56	8.40 × 10^−13^	−8.14	1.17 × 10^−10^	NEDD9	−2.57	9.84 × 10^−5^	−4.78	5.36 × 10^−5^
CCDC38	−6.14	1.57 × 10^−3^	−5.11	3.35 × 10^−2^	GDNF	−3.03	2.46 × 10^−5^	−7.19	1.05 × 10^−7^
TLE2	−2.01	1.68 × 10^−2^	−2.31	3.42 × 10^−2^	KCNE3	−4.26	1.62 × 10^−3^	−7.13	1.21 × 10^−4^
RBP5	−5.02	2.26 × 10^−5^	−3.96	4.70 × 10^−3^	WNT9A	−5.07	4.04 × 10^−5^	−6.09	8.89 × 10^−5^
C4orf50	−7.94	5.84 × 10^−9^	−4.14	1.17 × 10^−3^	PRG4	−5.32	1.37 × 10^−23^	−2.10	2.87 × 10^−4^
B3GALT2	−4.48	2.80 × 10^−3^	−5.51	3.56 × 10^−3^	COBL	−3.43	6.42 × 10^−3^	−6.04	7.06 × 10^−4^
TARBP2	−2.79	7.27 × 10^−38^	−2.06	3.69 × 10^−19^	APOBEC3G	−2.20	4.02 × 10^−5^	−7.67	8.91 × 10^−10^
Novelprotein	−6.46	9.07 × 10^−3^	−25.72	3.62 × 10^−22^	OAS1	−2.94	1.65 × 10^−9^	−3.42	8.78 × 10^−10^
FBLN2	−3.11	9.53 × 10^−47^	−2.81	1.67 × 10^−33^	DMD	−2.71	3.40 × 10^−3^	−3.79	8.03 × 10^−3^
B4GALNT1	−3.32	4.72 × 10^−16^	−2.03	1.15 × 10^−5^	ALDH3A1	−2.64	3.78 × 10^−65^	−8.13	1.41 × 10^−49^
PCSK9	−4.63	1.41 × 10^−4^	−6.83	5.09 × 10^−5^	UGT1A6	−2.76	1.51 × 10^−31^	−2.68	3.63 × 10^−26^
GPR143	−2.49	3.93 × 10^−27^	−2.19	2.95 × 10^−18^	MT1E	−4.79	4.34 × 10^−40^	−3.96	5.72 × 10^−25^
CCDC152	−2.34	2.79 × 10^−3^	−2.89	2.16 × 10^−3^	MGP	−3.62	2.83 × 10^−4^	−2.76	2.59 × 10^−2^
NPW	−3.41	9.72 × 10^−68^	−2.62	4.15 × 10^−37^	Novelprotein	−2.07	3.49 × 10^−2^	−3.45	8.85 × 10^−3^
H3-7	−6.10	1.02 × 10^−3^	−5.07	2.61 × 10^−2^	ICAM2	−3.50	5.84 × 10^−22^	−2.75	2.09 × 10^−12^
NCF2	−2.86	1.34 × 10^−3^	−4.26	2.00 × 10^−3^	PDCD6-AHRR	−23.70	3.17 × 10^−10^	−22.73	2.76 × 10^−8^
CDKN2A	−4.45	6.13 × 10^−163^	−2.22	8.92 × 10^−43^	ABHD14A-ACY1	−5.34	2.10 × 10^−4^	−5.60	2.04 × 10^−3^
INMT-MINDY4	−7.55	4.50 × 10^−7^	−3.30	2.84 × 10^−2^	KLHDC7B	−5.01	1.03 × 10^−3^	−3.98	3.73 × 10^−2^
ACTA2	−2.49	3.80 × 10^−11^	−5.63	4.69 × 10^−16^	ATP8B4	−3.84	1.94 × 10^−8^	−2.97	1.80 × 10^−4^
RAB6C	−3.79	3.20 × 10^−2^	−4.78	3.62 × 10^−2^	Novelprotein	−6.80	2.70 × 10^−5^	−4.32	3.34 × 10^−2^
MCF2L	−3.95	4.21 × 10^−7^	−3.17	5.57 × 10^−4^	LAMC3	−4.91	2.00 × 10^−35^	−2.55	1.88 × 10^−10^
TMEM37	−2.28	2.24 × 10^−2^	−2.69	4.78 × 10^−2^	LDLRAD4	−4.78	1.47 × 10^−6^	−2.16	4.02 × 10^−2^
ATP10A	−5.85	1.12 × 10^−4^	−6.26	2.77 × 10^−4^					
Novelprotein	−23.52	4.36 × 10^−10^	−22.52	3.90 × 10^−8^					
ARHGAP4	−3.16	3.42 × 10^−20^	−5.67	7.76 × 10^−32^					

Abbreviations: FC, fold change; DEGs, differentially expressed genes; H1792/Car, Car-resistant H1792 cell line; H1792/Pac, Pac-resistant H1792 cell line.

## Data Availability

Data may be provided with appropriate justification upon request.

## Supplementary Materials

The following supporting information can be downloaded at: https://www.mdpi.com/article/10.3390/cimb46120834/s1.
