# Supplementary material for: Transcriptomic Profiling of Carboplatin- and Paclitaxel-Resistant Lung Adenocarcinoma Cells Reveals CSF3 as a Potential Biomarker for the Carboplatin Plus Paclitaxel Doublet Regimens
Source: Curr Issues Mol Biol. 2024 Dec 11;46(12):13951–69. doi: 10.3390/cimb46120834 (PMC11727171; doi:10.3390/cimb46120834)
Supplement: Supplementary file 1 [file cimb-46-00834-s001.zip › cimb-3357083-supplementary.pdf]

(A)

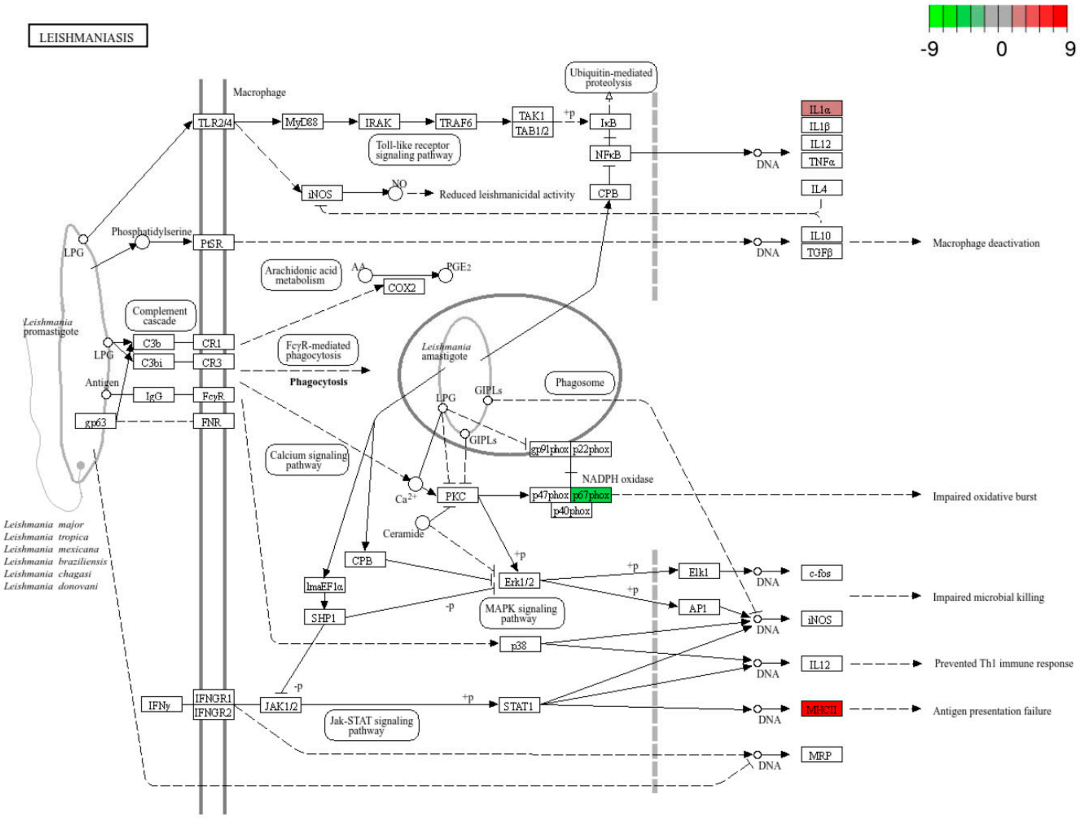
LEISHMANIASIS
-9
0
9
Macrophage
TLR2/4
MyD88
IRAK
TRAF6
TAK1
TAB1/2
IκB
NFκB
CPB
Ubiquitin-mediated proteolysis
Toll-like receptor signaling pathway
iNOS
NO
Reduced leishmanicidal activity
IL1α
IL1β
IL12
TNFα
IL4
IL10
TGFβ
DNA
Macrophage deactivation
LPG
Phosphatidylserine
PtSR
Arachidonic acid metabolism
AA
PGE2
COX2
Leishmania promastigote
Complement cascade
C3b
C3bi
CR1
CR3
FcγR-mediated phagocytosis
Phagocytosis
Antigen
IgG
FcγR
gp63
FNR
Leishmania amastigote
Phagosome
GIPLs
Calcium signaling pathway
gp91phox
p22phox
NADPH oxidase
Ca2+
PKC
p47phox
p67phox
p40phox
Impaired oxidative burst
Ceramide
Leishmania major
Leishmania tropica
Leishmania mexicana
Leishmania braziliensis
Leishmania chagasi
Leishmania donovani
CPB
Erk1/2
Elk1
c-fos
lmaEF1α
MAPK signaling pathway
AP1
Impaired microbial killing
SHP1
iNOS
p38
IL12
Prevented Th1 immune response
IFNγ
IFNGR1
IFNGR2
JAK1/2
Jak-STAT signaling pathway
STAT1
MHCII
Antigen presentation failure
MRP

(B)

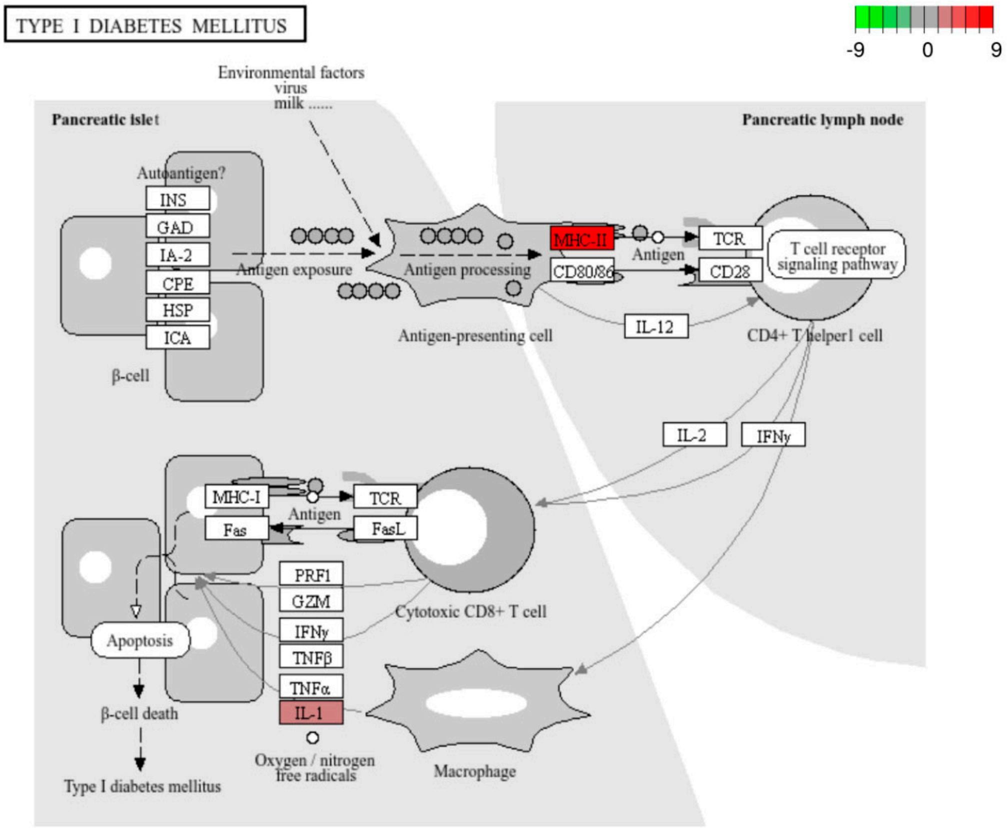
TYPE I DIABETES MELLITUS
-9
0
9
Environmental factors
virus
milk ......
Pancreatic islet
Pancreatic lymph node
Autoantigen?
INS
GAD
IA-2
CPE
HSP
ICA
β-cell
Antigen exposure
Antigen processing
MHC-II
CD80/86
Antigen
TCR
CD28
T cell receptor signaling pathway
Antigen-presenting cell
IL-12
CD4+ T helper1 cell
IL-2
IFNγ
MHC-I
Fas
Antigen
TCR
FasL
PRF1
GZM
IFNγ
TNFβ
TNFα
IL-1
Cytotoxic CD8+ T cell
Apoptosis
β-cell death
Type I diabetes mellitus
Oxygen / nitrogen free radicals
Macrophage

(C)

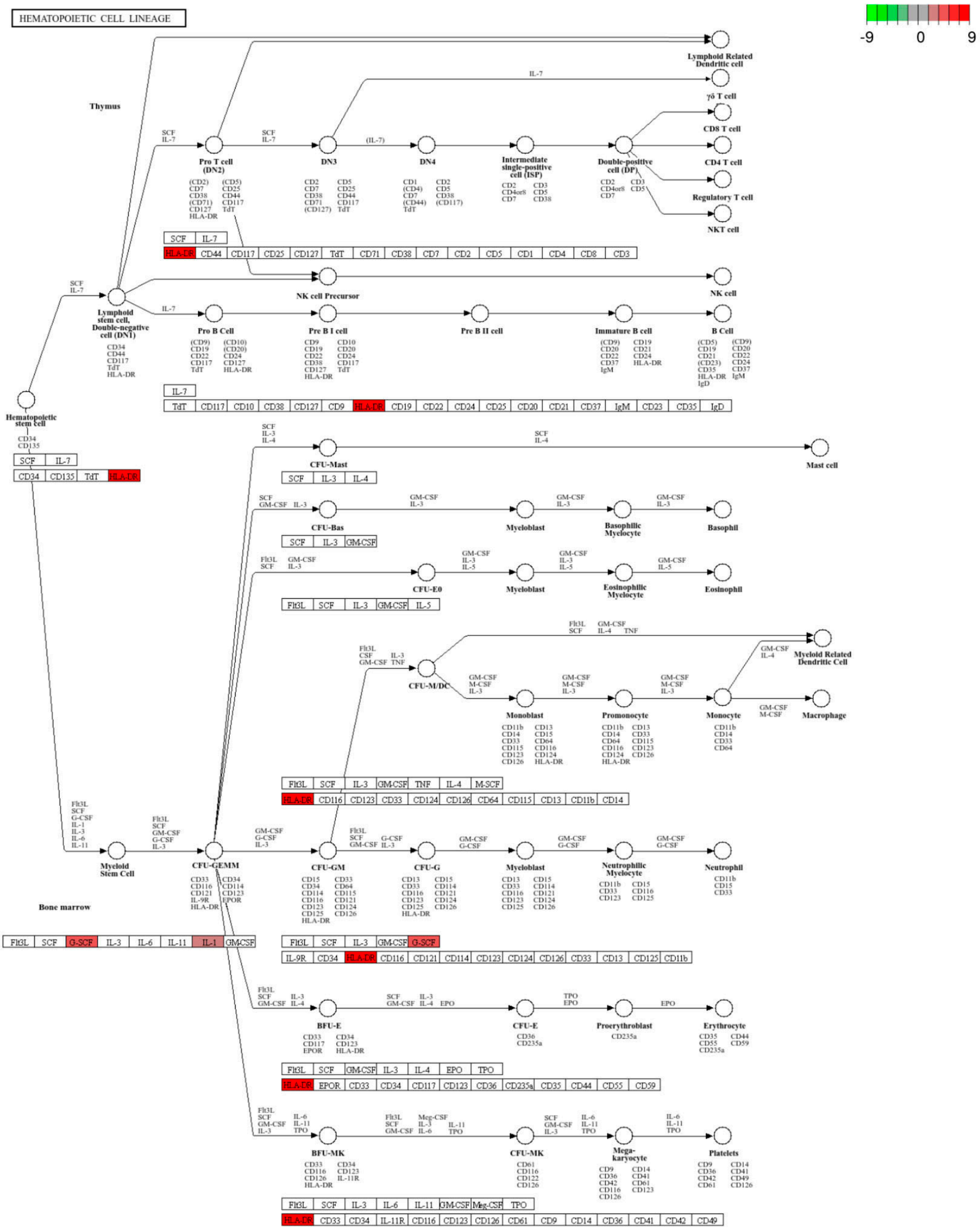
HEMATOPOIETIC CELL LINEAGE
-9 0 9
Thymus
Bone marrow
Hematopoietic stem cell
CD34
CD135
SCF IL-7
CD34 CD135 TdT HLA-DR
Lymphoid stem cell, Double-negative cell (DN1)
CD34
CD44
CD117
TdT
HLA-DR
Pro T cell (DN2)
(CD2) (CD5)
CD7 CD25
CD38 CD44
(CD71) CD117
CD127 TdT
HLA-DR
DN3
CD2 CD5
CD7 CD25
CD38 CD44
CD71 CD117
(CD127) TdT
DN4
CD1 CD2
(CD4) CD5
CD7 CD38
(CD44) (CD117)
TdT
Intermediate single-positive cell (ISP)
CD2 CD3
CD4or8 CD5
CD7 CD38
Double-positive cell (DP)
CD2 CD3
CD4or8 CD5
CD7
Lymphoid Related Dendritic cell
γδ T cell
CD8 T cell
CD4 T cell
Regulatory T cell
NKT cell
SCF IL-7
HLA-DR CD44 CD117 CD25 CD127 TdT CD71 CD38 CD7 CD2 CD5 CD1 CD4 CD8 CD3
NK cell Precursor
NK cell
Pro B Cell
(CD9) (CD10)
CD19 (CD20)
CD22 CD24
CD117 CD127
TdT HLA-DR
Pre B I cell
CD9 CD10
CD19 CD20
CD22 CD24
CD38 CD117
CD127 TdT
HLA-DR
Pre B II cell
Immature B cell
(CD9) CD19
CD20 CD21
CD22 CD24
CD37 HLA-DR
IgM
B Cell
(CD5) (CD9)
CD19 CD20
CD21 CD22
(CD23) CD24
CD35 CD37
HLA-DR IgM
IgD
IL-7
TdT CD117 CD10 CD38 CD127 CD9 HLA-DR CD19 CD22 CD24 CD25 CD20 CD21 CD37 IgM CD23 CD35 IgD
CFU-Mast
Mast cell
SCF IL-3 IL-4
CFU-Bas
Myeloblast
Basophilic Myelocyte
Basophil
SCF IL-3 GM-CSF
CFU-E0
Myeloblast
Eosinophilic Myelocyte
Eosinophil
Flt3L SCF IL-3 GM-CSF IL-5
CFU-M/DC
Myeloid Related Dendritic Cell
Monoblast
CD11b CD13
CD14 CD15
CD33 CD64
CD115 CD116
CD123 CD124
CD126 HLA-DR
Promonocyte
CD11b CD13
CD14 CD33
CD64 CD115
CD116 CD123
CD124 CD126
HLA-DR
Monocyte
CD11b
CD14
CD33
CD64
Macrophage
Flt3L SCF IL-3 GM-CSF TNF IL-4 M-SCF
HLA-DR CD116 CD123 CD33 CD124 CD126 CD64 CD115 CD13 CD11b CD14
Myeloid Stem Cell
CFU-GEMM
CD33 CD34
CD116 CD114
CD121 CD123
IL-9R EPOR
HLA-DR
CFU-GM
CD15 CD33
CD34 CD64
CD114 CD115
CD116 CD121
CD123 CD124
CD125 CD126
HLA-DR
CFU-G
CD13 CD15
CD33 CD114
CD116 CD121
CD123 CD124
CD125 CD126
HLA-DR
Myeloblast
CD13 CD15
CD33 CD114
CD116 CD121
CD123 CD124
CD125 CD126
Neutrophilic Myelocyte
CD11b CD15
CD33 CD116
CD123 CD125
Neutrophil
CD11b
CD15
CD33
Flt3L SCF G-SCF IL-3 IL-6 IL-11 IL-1 GM-CSF
Flt3L SCF IL-3 GM-CSF G-SCF
IL-9R CD34 HLA-DR CD116 CD121 CD114 CD123 CD124 CD126 CD33 CD13 CD125 CD11b
BFU-E
CD33 CD34
CD117 CD123
EPOR HLA-DR
CFU-E
CD36
CD235a
Proerythroblast
CD235a
Erythrocyte
CD35 CD44
CD55 CD59
CD235a
Flt3L SCF GM-CSF IL-3 IL-4 EPO TPO
HLA-DR EPOR CD33 CD34 CD117 CD123 CD36 CD235a CD35 CD44 CD55 CD59
BFU-MK
CD33 CD34
CD116 CD123
CD126 IL-11R
HLA-DR
CFU-MK
CD61
CD116
CD122
CD126
Mega-karyocyte
CD9 CD14
CD36 CD41
CD42 CD61
CD116 CD123
CD126
Platelets
CD9 CD14
CD36 CD41
CD42 CD49
CD61 CD126
Flt3L SCF IL-3 IL-6 IL-11 GM-CSF Meg-CSF TPO
HLA-DR CD33 CD34 IL-11R CD116 CD123 CD126 CD61 CD9 CD14 CD36 CD41 CD42 CD49

(D)

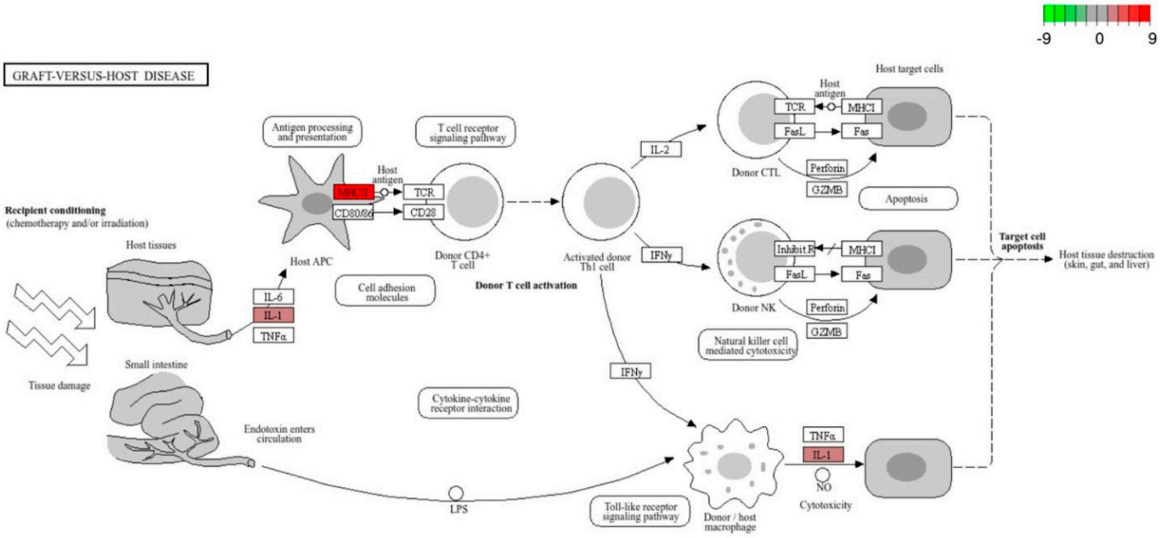

Figure S1. KEGG pathway enrichment analysis of common DEGs (Co-DEGs) in drug-resistant cell lines. (A) Leishmaniasis. (B) Type I diabetes mellitus. (C) Hematopoietic cell lineage. (D) Graft-versus-host disease.
